# First experimental insights into the ex situ cultivation of *Seseli Resinosum* in vertical gardens

**DOI:** 10.1038/s41598-026-41230-x

**Published:** 2026-02-27

**Authors:** Nermin Başaran, Sümeyra Elmastaş, Engin Eroğlu

**Affiliations:** Department of Landscape Architecture, Düzce University, Düzce, Türkiye Turkey

**Keywords:** Ex situ cultivation, Düzce, Endemic plant, *Seseli resinosum*, Vertical garden, Ecology, Ecology, Plant sciences

## Abstract

Vertical garden systems represent an innovative approach that simulates rocky habitats, which are often characterized by high species diversity and endemism. In this study, we experimentally evaluated the performance of two vertical garden systems as an ex situ conservation strategy to ensure the sustainability of Seseli resinosum, a local endemic species of Düzce Province. In the first phase of this four-stage study, S. resinosum was monitored in its natural ecosystem for one vegetation period, and relevant data were collected. In the second phase, the species was collected and cultivated under controlled conditions. During the third and fourth phases, individuals were transplanted into two different vertical garden modules, where subsequent monitoring was conducted. Ecological trait analyses revealed that S. resinosum exhibits strong similarities with rocky-habitat species such as Hypericum perforatum, Silene italica, and Scabiosa columbaria, and Origanum vulgare. The results demonstrated that felt-based systems are unsuitable for the ex situ conservation of S. resinosum due to insufficient moisture retention and thermal buffering. In contrast, pot-based vertical garden systems supported successful vegetative and reproductive development. These findings indicate that modular pot-based vertical systems should be preferred over felt-based designs for the ex situ conservation of endemic drought-tolerant species under comparable changing climatic conditions.

## Introduction

 The IUCN Red List includes over 1172,600 assessed species of animals, plants, and fungi as of July 2024^1^. Approximately 28% of all evaluated species are classified as threatened with extinction, categorized as Vulnerable (VU), Endangered (EN), and Critically Endangered (CR)^2^. This global decline highlights the urgency of implementing effective conservation strategies to safeguard biodiversity and maintaining ecosystem functioning^3^. In situ conservation focuses on preserving biodiversity within natural habitats^4,5^, whereas ex situ conservation involves safeguarding biodiversity outside of its natural habitats^6,7^. Although in situ conservation is widely regarded as the primary approach for maintaining viable populations, especially local endemics^8,9^, ex situ strategies can complement these efforts by providing insurance populations for rare or highly threatened species^10,11^.

Endemic species represent irreplaceable components of regional biodiversity, contributing uniquely to the ecological structure and evolutionary distinctiveness of their native habitats^12,13^. However, urbanization, climate change and land-use change have intensified pressures on biodiversity, disproportionately affecting taxa with restricted geographic distributions^14–16^. Species confined to narrow ecological niches or limited spatial ranges are especially vulnerable to habitat loss and fragmentation^15^. Endemic species can be categorized according to their spatial distribution, ranging from strictly local endemics to provincial and regional endemics^17^. Regardless of scale, their restricted occurrence increases extinction susceptibility compared to more widely distributed species. Consequently, integrated conservation frameworks that combine in situ protection with complementary ex situ measures are increasingly recognized as essential for sustaining endemic plant diversity^18,19^. In countries with high habitat diversity and biodiversity, such as Türkiye, the combined application of in situ and ex situ conservation methods is widely recognized as an effective strategy^20,21^.

Türkiye is recognized as a biodiversity hotspot due to its unique geographical position^22^. The country hosts approximately 12,000 plant taxa, ranking ninth in Europe in terms of floristic diversity^23^. With nearly 3,000 endemic species, Türkiye exhibits one of the highest endemism rates in the region^14,24^. Endemic species are particularly concentrated in Anatolia, especially within mountainous landscapes that promote habitat heterogeneity and ecological isolation^25^. Düzce Province represents a floristically diverse area characterized by 102 plant families and approximately 1200 species and subspecies^24^. The region encompasses multiple vegetation types, including dune systems, maquis formations, rocky habitats, riparian vegetation, forest ecosystems, and alpine communities^26^. Notably, the region hosts 14 rare and 66 endemic plant species^27^. According to IUCN criteria, a total of 12 species in Düzce are considered “threatened,” including those categorized as Critically Endangered (CR)^28^. The presence of threatened and range-restricted taxa highlights the conservation importance of the region and the need for coordinated management efforts.

Many studies have examined both in situ conservation methods^29^, and ex situ conservation approaches^30^, particularly for endemic plant species categorized as CR or Endangered EN. In ex situ conservation, plant species are preserved in the form of seeds, tubers, tissue explants, pollen, or DNA samples, either in field gene banks, living collections within botanical gardens and arboreta, or under controlled artificial storage conditions^31,32^. Although a variety of techniques are employed to protect threatened taxa, ex situ plant conservation programs have traditionally emphasized seed banking^33^. This approach has been favored because it offers a relatively simple and cost-effective method for long-term germplasm preservation with minimal maintenance requirements^34,35^. However, seed banking is not feasible for all species, as some possess recalcitrant seeds or demand complex storage protocols^33,34^. Among alternative ex situ conservation strategies, plant tissue culture has become the most widely adopted method owing to its versatility and effectiveness^11^. In ex situ conservation efforts, botanical gardens play a crucial role in safeguarding and propagating species by recreating habitats that closely resemble their natural conditions. Beyond botanical gardens, the ex situ conservation of endangered species through collection and propagation is also being implemented in public green spaces and even in private gardens^36^. The effectiveness of such efforts depends largely on understanding species-specific biotic and abiotic requirements, as well as habitat preferences, which are essential for designing effective conservation strategies^37,38^. In botanical gardens, artificial environments established for plant growth, survival, and reproduction are carefully designed to approximate the biotic and abiotic conditions of natural habitats^39^. For instance, in collections designed to conserve and display species adapted to rocky environments, habitat-specific features such as rock formations are deliberately incorporated into the design^40^. By contrast, such habitat-oriented approaches are not consistently applied in public green spaces and private gardens^41^.

The Vertical Garden System (VGS) has emerged as an innovative nature-based system (NBS) that mimics rocky habitats, which support high levels of species diversity and endemism^42^. While most VGS research has focused on ornamental^43^, architectural^44,45^, or energy-efficiency^46,47^ functions^48^, its application in plant conservation remains insufficiently explored. Only a limited number of studies have introduced native species to enhance biodiversity^49,50^. Moreover, explicit assessments of VGSs as structured ex situ habitats remain scarce in the literature^51^. Previous research has demonstrated that plant performance varies across vertical garden systems, as structural configurations and substrate depth significantly influence species establishment and adaptation^52^. In addition, VGS designs differ in durability and operational lifespan, which may further affect long-term cultivation success^53,54^. VGS are generally classified as covered, soil, or sheet-based^48^. In modular systems, pots replace the felt-based growing medium^55^. This study addresses this gap by evaluating the potential of vertical garden systems as alternative ex situ conservation habitats. We selected *Seseli resinosum* Freyn & Sint. an endemic species of Düzce Province naturally adapted to rocky substrates, as a model taxon due to its restricted distribution and habitat specialization. Given that rocky environments are characterized by shallow soils, high drainage, and structural heterogeneity, VGSs may provide a functional analogue when appropriately designed. Within this framework, the following hypotheses were formulated and tested:

(i) assess the survival and adaptation capacity of *S. resinosum* under different VGS designs;
(ii) determine how structural and substrate characteristics influence *S. resinosum* performance; and.
(iii) evaluate the potential of VGSs as a complementary ex situ conservation strategy for rocky-habitat endemic species.

## Materials and methods

### Conceptual framework

This study focuses on ex situ conservation for ensuring the sustainability of *S. resinosum* an endemic species of Düzce Province, in the face of changing climate and environmental conditions. The research design is structured around four main stages. In the first stage of the study, fieldwork was conducted to identify the habitat characteristics, soil properties, and associated plant species of *S. resinosum.* We collected soil samples from the field at a depth of 0–30 cm. Additionally, we identified and documented plant species co-occurring with *S*. *resinosum.* Any unidentified species were collected and pressed for examination for taxonomic classification. Climatic data were obtained from the Düzce Provincial Directorate of Meteorology. In the second stage, specimens of *S. resinosum* were collected and cultivated for approximately 5 months in pots at the greenhouse of the Faculty of Forestry, Düzce University. In the third stage, the plants were transferred to two different VGSs where monitoring studies were continued. The main rationale for this is that several studies in the literature emphasize differences in plant development across various VGSs. In the final stage of the study, we monitored both abiotic and biotic factors to evaluate the growth dynamics of the species during the vegetation process. The data obtained at the end of the study were compared with the natural habitat. As a result of this evaluation, conclusions were reached about the ability of the species to adapt to different growing conditions.

### Material and study area

**Fig. 1:**
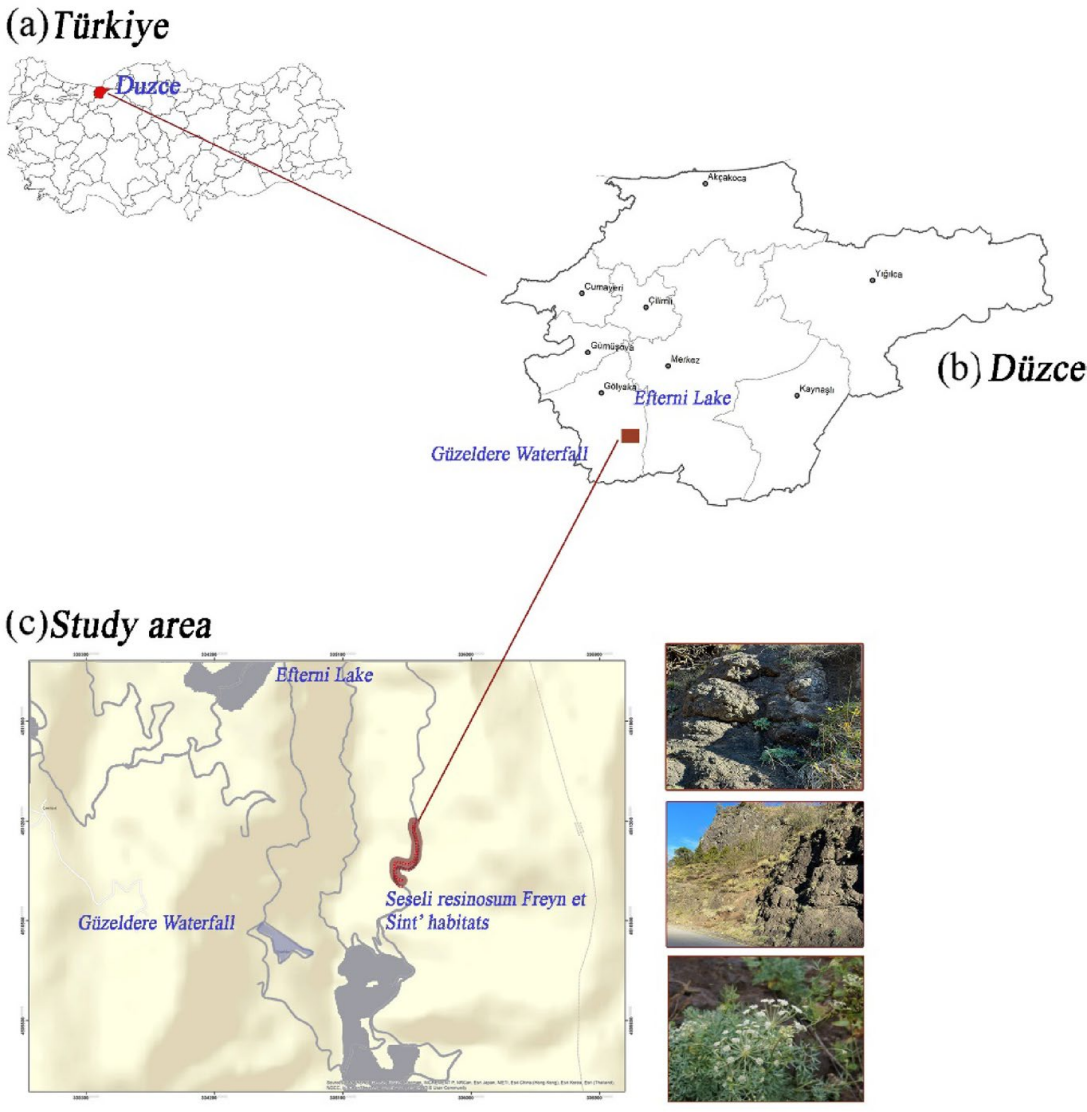
Geographic location and habitat of *S. resinosum* in Düzce, Türkiye. **a** Overview map showing the location of Düzce province within Türkiye. **b** Regional map of Düzce province highlighting the location of Efteni Lake and Güzeldere Waterfall. **c** Detailed study area map indicating the specific habitat of S. resinosum between Efteni Lake and Güzeldere Waterfall. Photographs on the right display the typical rocky habitat and vegetation where the species is found.

The principal material of this study is *S. resinosum* an endemic plant taxon restricted to Düzce Province, where monitoring studies are currently ongoing. The formal identification of *S. resinosum* was originally undertaken by the botanists Josef Franz Freyn and Paul Ernst Emil Sintenis, who first described the species. Voucher specimens of *S. resinosum* are currently deposited and exhibited in the Herbarium of Düzce University, Turkey. *S. resinosum* is characterized by distinctive morphological features^56^. Its most defining traits include glabrous leaves, lanceolate to oblong-lanceolate leaf segments, and short umbels with rays measuring between 17 and 33 mm^57^. The species typically inhabits limestone cliffs, mixed woodlands, and rocky environments (Fig. 1), particularly in coastal areas. It occurs at elevations up to 600 m above sea level and flowers between July and August^56^.

Its distribution is limited to the provinces of Zonguldak, Bartın, Kastamonu, and Düzce in Türkiye. The plant material of species used in this study was collected from Düzce Province. *S. resinosum* is found within a 1.5 km-long rocky habitat and landslide-prone areas located within the boundaries of Düzce Central District^28^. *S. resinosum* occurs within plant communities containing complementary taxa in its natural habitat; however, the degree of vegetation cover and overlap remains relatively low^56^. According to Turkish State Meteorological Service (TSMS) data, the study area receives an average annual precipitation of approximately 833 mm, with a mean annual temperature of 13.1 °C^58^. Although the native soil in the habitat of *S. resinosum* is classified as red-brown forest soil, it is characterized by a low organic matter content and the presence of an E horizon depleted in iron (FE). This pedogenic condition is likely a consequence of the plant’s location on steep slopes within a documented landslide zone. The effective soil depth in this area was measured at approximately 5 cm. Soil pH ranges from 6.2 to 6.8, indicating a moderately acidic profile.

### Method

#### Field Studies and Observation

The most suitable period for vegetation sampling studies in Düzce is between April and October, as this corresponds to the active growing season and provides optimal conditions for sampling^14^. Conversely, the optimal period for plant collection is between November and March. Within the scope of this study, fieldwork was conducted in two phases: plant collection during February–March and vegetation sampling between April and October. Vegetation surveys were conducted using the Braun-Blanquet method^59^, which is based on ecological parameters and employed to establish and assess the sample plots. Sampling was conducted using the 1 × 1 m quadrat method. We classified unidentified species in the field at the Herbarium of the Faculty of Forestry, Düzce University.

#### Collecting and cultivation *S. resinosum*

Specimens of *S. resinosum* collected from Düzce Province, Gölyaka District, Bıçkı Düzü locality, rocky habitat (625 m above sea level, at coordinates N 40°43.607′, E 031°03.107′- WGS84) in November. Since this species is not included among the taxa legally prohibited from being collected for scientific purposes, no special permit was required for its collection. Although *S. resinosum* is an endemic plant species, it is not listed in the IUCN Red List database. Similarly, according to the National Biodiversity Database of Türkiye (Nuh’un Gemisi), managed by the Ministry of Agriculture and Forestry, no threat category has been assigned to this species. Therefore, our research and field studies were conducted in full compliance with institutional and national guidelines^60^. In addition, this research was carried out within the framework of a The Scientific and Technological Research Council of Türkiye (TÜBİTAK), funded research project (project number: 1919B012219240). Collecting plant material other than those species prohibited from being collected in the Official Gazette, for scientific purposes within the scope of TÜBİTAK-approved research projects, does not constitute a violation of the relevant legislation. The specimens were cultivated in a growing medium composed of perlite, peat, and native soil mixed in a 1:1:1 ratio. Standardized commercial growing medium was not used in this study. Instead, emphasis was placed on replicating natural soil conditions to enhance ecological compatibility.

#### Establishment of VGS and plant transplantation

**Fig. 2:**
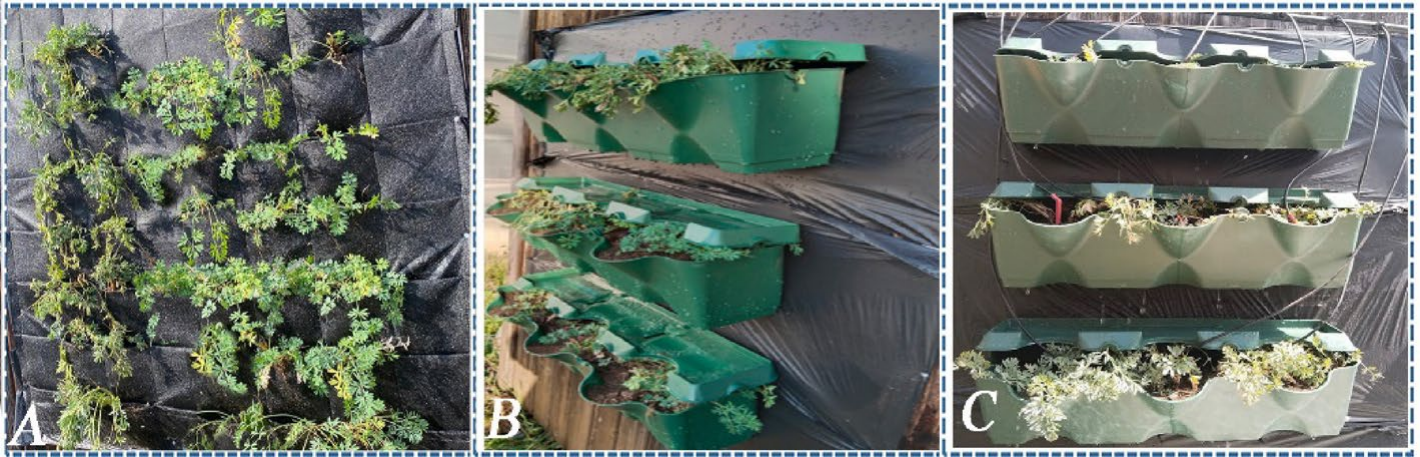
VGS prototypes designed for ex-situ cultivation. **A** Felt-based system displaying individual planting pockets with S. resinosum specimens. **B** Multi-tier pot-based system with modular plastic containers affixed to a vertical surface. C Pot-based system vertical arrangement featuring horizontal planter rows with irrigation setup.

Plants grown in pots from early November to the end of March, and they were planted in VGS modules prepared at the end of March. Before transplantation, root and stem lengths were measured to establish baseline data for comparing growth rates across the two VGS. In this study, two vertical garden modules, each measuring 1 × 1 m, were constructed. Of these, the first employed a felt-based system, while the second used a pot-based system. In this study, the primary rationale for employing two different types of VGSs lies in the absence of a standardized design framework, which results in variations in the amount of growing media as well as differences in water and moisture requirements between systems. One of the main objectives, therefore, is to determine which VGS type provides the most suitable growing conditions for *S. resinosum.* Both modules were installed on the southwest façade, considering the site’s spatial orientation and landscape view. Our felt systems utilize felt pockets as the growth medium and typically include seven fundamental components: structural support, supporting elements, growth medium, irrigation and drainage systems, fogging system, and plants. In some cases, lighting can be added if needed. In this study, we provided natural light (Fig. 2).

Carrier profiles were constructed using galvanized metal, while polyvinyl chloride (PVC) panels were employed as moisture-resistant support elements. To maintain adequate moisture levels in felt-based systems, 2 mm perforations were placed at 10 cm intervals, and polyurethane plastic tubes were inserted. Irrigation was applied using a controlled drip irrigation system, and water supply was adjusted according to seasonal climatic conditions and plant performance. The misting system was deemed unnecessary for the present study. A drip irrigation system was installed in both modules, configured similarly and programmed to operate once or twice daily depending on air temperature. During transplantation, damaged roots were pruned while preserved the surrounding soil of plants and as much as possible to support acclimation. Following planting, initial irrigation was applied to ensure root establishment. The substrate used in the VGSs consisted of a 1:1:1 mixture of perlite, peat, and natural soil, identical to the medium used during plant propagation. Plants were randomly assigned to the felt-based and pot-based systems.

#### Monitoring and data analysis

In the plant monitoring study, biotic and abiotic factors were first recorded both in the natural habitat and in the area where the research design was established. Table 1 presents the measured biotic and abiotic parameters.

**Table 1:** Descriptions of the measuring biotic and abiotic factors.

Factors	Parameter	Natural habitat /VGSs Environment(April- October)
Abiotic	Light	Light intensity (lux)
	Air temperature	Air temperature (°C)
	Soil temperature	Volumetric soil moisture (mm)
	Soil moisture	Volumetric soil moisture (mm)
	Precipitation	Amount of Precipitation (mm)
Biotic	Plant height	Height from the soil surface to the apex of the flowering part (cm)
	Pedicel Length	Length of the stalk connecting an individual flower to the inflorescence axis (cm)
	Number of leaves	Total count of fully developed leaves per individual.
	Root length	Measured from the root collar to the tip of the longest root (cm).
	First blooming time	Date of first flowering
	Last blooming time	Date of last flower senescence

Table 1. Descriptions of the measuring biotic and abiotic factors.

The abiotic parameters monitored in this study comprised light intensity, air temperature, soil temperature, soil moisture, and precipitation. Photosynthetically active radiation was quantified using multiple light sensors positioned natural habitat and VGSs environment. Measurements were taken simultaneously between 11:00 and 14:00 under comparable conditions. In order to determine the amount of precipitation, samples were collected using 500 mL volume polyethylene bottles connected to funnels with 100 cm² mouth openings for the observation of precipitation. Abiotic factors were monitored at monthly intervals between March and October in identical sampling plots, and the resulting records were compiled into the dataset. Abiotic factors were measured weekly. The arithmetic mean of the obtained data was calculated to derive average values, whereas precipitation was evaluated based on monthly total precipitation.

Due to the species’ limited spatial distribution and regional constraints, only a small number of specimens could be collected. Therefore, inferential statistical tests were not applied, and the analysis was based on descriptive and comparative approaches. Accordingly, instead of conducting monthly measurements, root and leaf assessments were limited to the beginning and end of the vegetation period. In this context, the dimensions of the plant were assessed by measuring two perpendicular widths and the height at a 90-degree angle. A plant index was calculated by taking the arithmetic mean of these values. Additional observations involved photographic documentation of the growth process and systematic recording of the developmental stages of *S. resinosum.* Specimens cultivated within the VGS modules. In addition, we conducted ecological trait similarity analysis to reveal the similarities of ecological traits with species in the natural ecosystem. In this context, Jaccard Similarity (suitable for binary data) was calculated for species pairs. The Jaccard index is a statistic used to measure the similarity of samples^61^.

1 $$\:{D}_{j}\left(A,B\right)=1-J(A,B)$$

where J(A, B) denotes the Jaccard index, and A and B represent the sets being compared. To visualize the index, we used Python 3.11, NumPy 1.26, Pandas 2.2, SciPy 1.11, Matplotlib 3.8.

## Results

### Natural habitat assessment of *S. resinosum* in the düzce

Field observations revealed that *S. resinosum* predominantly grows in colonies, particularly in rocky habitats. While individuals were frequently encountered in dense populations within their natural habitats, solitary occurrences were also recorded. The herbaceous, semi-woody, and shrub species co-occurring with *S. resinosum* within its 1 km² distribution area are listed in Table 2. Additionally, the heatmap of standardized ecological traits.

**Table 2:** Plant species co-occurring with *S. resinosum* Freyn & Sint. in natural habitat.

No	Species	Family	Life Form	Habitat	Flowering Period	Ecological Tolerance	Distribution of Türkiye
1	*Alyssum murale* WALDST. ET KIT.	Brassicaceae	Perennial	Rocky slopes, Dry scrublands	4–7	Drought, Nutrient-poor	N., W., S., O. and E. Anatolia
2	*Asplenium trichomanes* L.	Aspleniaceae	Perennial	Rocky slopes, Cliffs	….	Shade-tolerant, Cold-hardy	External Anatolia
3	*Briza maxima* L.	Poaceae	Annual	Dry scrublands, Roadsides, Ruderal areas	4–5	Drought, UV stress	Trakya, External Anatolia
4	*Campanula persicifolia* L.	Campanulaceae	Perennial	Deciduous forests, Calcareous soils, Roadsides	6–8	Tolerates partial shade	NW. Türkiye
5	*Convolvulus cantabrica* L.	Convolvulaceae	Perennial	Rocky slopes,, Roadsides, Ruderal areas, Grasslands	4–8	Drought, Nutrient-poor	External Anatolia
6	*Clematis vitalba* L.	Ranunculaceae	Perennial	Riparian zones, Open woodlands, Cliffs, Rocky slopes	6–8	Poor fertility, Moderate drought	External Anatolia
7	*Dactylis glomerata* L.	Poaceae	Perennial	Roadsides, Ruderal areas, Open grasslands, Woodland edge	5–7	Cold, Drought	NW. Türkiye, NE., S. and SE. Anatolia
8	*Dianthus colocephalus* Boiss	Caryophyllaceae	Perennial	Rocky slopes	5–9	UV stress	W., N., O. and E. Anatolia
9	*Euphorbia amygdaloides* L.	Euphorbiaceae	Perennial	Broadleaf woodlands	3–8	Moderate drought, Cold	NW. Türkiye, N. Anatolia
10	*Euphorbia stricta* L.	Euphorbiaceae	Annual	Dry grasslands, Rocky slopes, Calcareous soils, Roadsides,	4–8	Drought	Trakya, External Anatolia
11	*Galium paschale* FORSSKAL	Rubiaceae	Perennial	Broadleaf woodlands	6–8	Shade-tolerant	N., SW. and Inland Anatolia
12	*Hedera helix* L.	Araliaceae	Perennial	Broadleaf and Mixed woodlands	8–9	Shade-tolerant	Trakya, External Anatolia
13	Hypericum perforatum L.	Clusiaceae	Perennial	Grasslands, Rocky slopes, Roadsides, Ruderal areas	5–8	UV stress, Drought, Nutrient-poor	N. and W. Anatolia
14	Origanum vulgare L.	Lamiaceae	Perennial	Rocky slopes, Open woodlands, Dry scrublands	4–6	Drought, Nutrient-poor	NW. And Continental Anatolia
15	Salvia forskahlei L.	Lamiaceae	Perennial	Calcareous soils, Woodlands	3–5	Moderate drought, Cold	N. Anatolia
16	Scabiosa columbaria L.	Dipsacaceae	Perennial	Calcareous soils, Limestone grasslands	6–9	Drought, Nutrient-poor	N. Anatolia
17	Saxifraga rotundifolia L.	Saxifragaceae	Perennial	Rocky slopes, Calcareous soils, Cliffs	6–9	Shade-tolerant	N. Türkiye
18	Silene italica L.	Caryophyllaceae	Perennial	Rocky slopes, Dry scrubland, Open grasslands,	6–7	Drought, Nutrient-poor	W., N., O. and E. Anatolia
19	*Tanacetum parthenium* (L.) Sch.Bip.	Asteraceae	Herbaceous	Rocky slopes, Roadsides, Ruderal areas	5–9	Moderate drought, Cold	Trakya, External Anatolia
20	*Verbascum blattaria L.*	Scrophulariaceae	Biennial	Ruderal areas, Roadsides, Grasslands, Woodland edges	4–9	Drought, Cold, Fire/Disturbance	N. Anatolia

Table 3. Plant species co-occurring with *S. resinosum* in natural habitat.

**Fig. 3:**
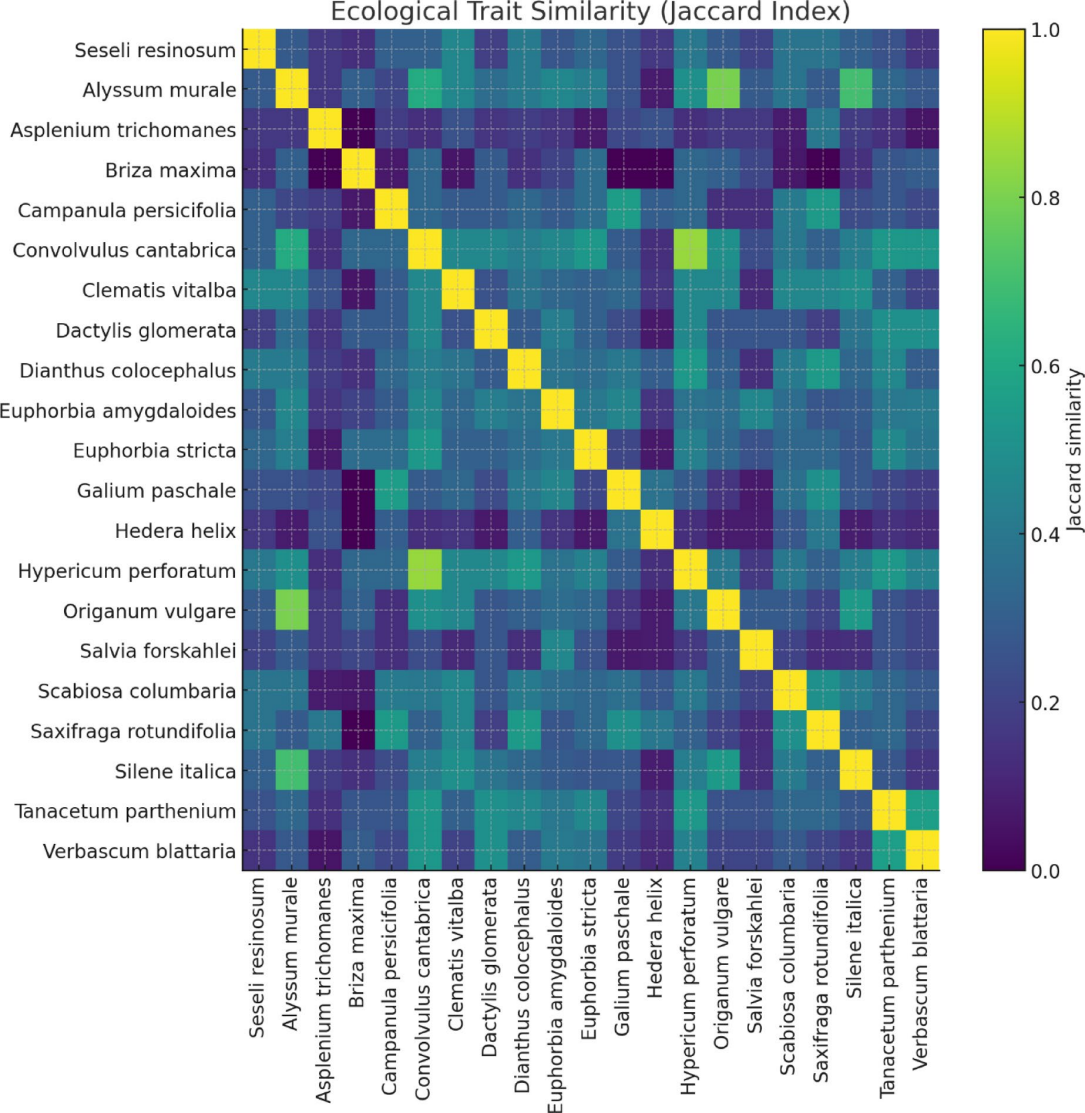
Heatmap of standardized ecological traits. *Heatmap illustrates pairwise similarity values (0–1)*,* where higher values (yellow) indicate stronger overlap in ecological traits and lower values (blue–purple) represent weaker similarity.*

Although *S. resinosum* does not form a dense plant assemblage, it was recorded in co-occurrence with 20 taxa representing 17 families along the 1.5 km transect for proper VGSs. Table 3; Fig. 3 reveal that *S. resinosum* shares ecological characteristics with species tolerant to drought, nutrient-poor conditions, and UV stress. In addition, the tolerance of these species to drought and nutrient-poor contents has shown that they are also suitable for VGS. It is particularly similar to species preferring calcareous rocky habitats, such as *Hypericum perforatum* L., *Origanum vulgare* L., *Silene italica* L., and *Scabiosa columbaria* L. However, it is distinctly different from the shade-tolerant *Hedera helix* L. and *Galium paschale* Forssk., or the ruderal-adapted species *Briza maxima* L. and *Tanacetum parthenium* (L.) Sch. Bip.

### Assessment of *S. resinosum* adaptation to different VGS

Key morphological and physiological traits, such as root architecture, leaf structure, and growth rate, serve as critical indicators of a plant’s adaptability to environmental stressors and site-specific conditions^28^. In the early stages of the experiment, particularly during the relatively humid months of March and April, *S. resinosum* exhibited greater early-stage growth in the felt-based system, achieving approximately 60–70% surface coverage, compared to about 40–50% in the pot-based system. By May, as mean air temperature increased from 13.2 °C in April to 17 °C, and the frequency of consecutive dry days increased, an automated irrigation system was activated to compensate for emerging moisture deficits.

**Fig. 4:**
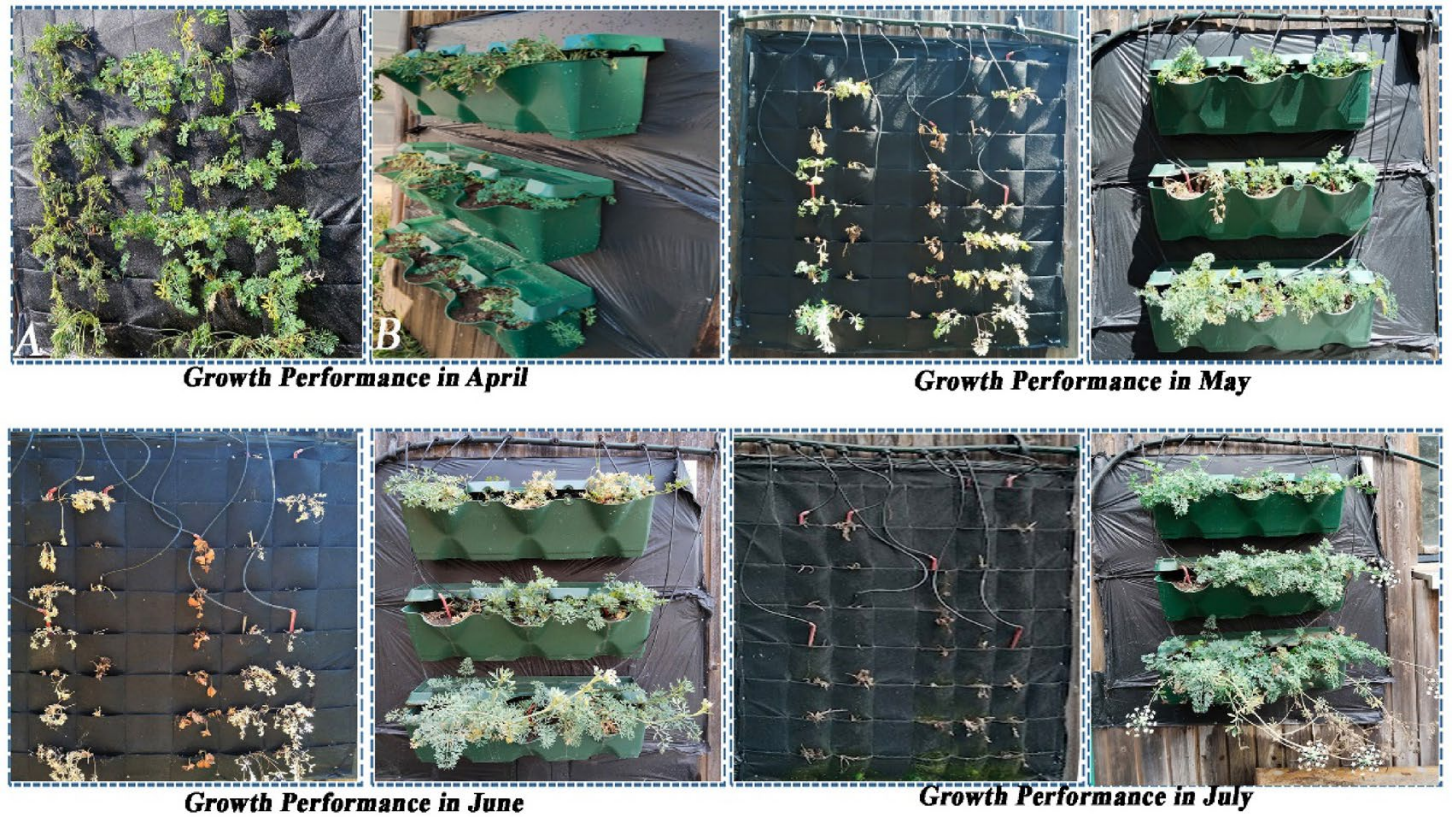
Growth performance in different months (A: Felt-based system; B: Pot-based system). *Caption: Monthly growth performance of S. resinosum in two vertical cultivation systems*,* (A) felt-based and (B) Pot-based system*,* between April and July. The felt-based system shows a progressive decline in plant vitality*,* with significant dieback observed by June and near-complete desiccation in July. In contrast*,* the pot-based system maintains healthier and more vigorous growth throughout the period*,* with lush foliage and flowering evident in July.*

By mid-May, distinct differences in water retention and irrigation demands became apparent between the two systems. Although both systems were irrigated at equal frequency, the pot-based system required watering once daily for 5 min, while the felt-based system necessitated 2 irrigation cycles per day of the same duration. After irrigation (09.00 am), soil moisture in the pot-based system remained relatively stable (42–45%), whereas in the felt-based system it decreased to ~ 40% within three hours. Additionally, substrate temperature in the felt system exceeded that of the pot-based system by 2–3 °C. As temperatures continued to climb in June, evaporation rates in the felt system increased sharply, rendering the initial irrigation regime inadequate to sustain optimal moisture levels. In response, the irrigation frequency for the felt system was increased to twice daily with sessions extended to 5–10 min. Additionally, irrigation times were shifted to the early morning (08.00 am) and late evening (07.00 pm) hours to minimize water loss through evaporation. Despite these adjustments, the felt substrate dried out approximately every 2 h, leading to a rapid decline in substrate humidity. This extreme desiccation resulted in the complete wilting and death of all *S. resinosum* individuals within the felt system approximately 3.5 months after initial planting (Fig. 4).

Figure 4 comparisons depict plant development in April, May, June, and July, illustrating system-dependent differences in vegetative vigor and survival across the growing season. The felt-based module, when implemented in open-air conditions using native habitat soil, yielded unsatisfactory results. However, in modular pot systems with an 8 cm soil depth, the species exhibited a vegetation cycle comparable to that observed in its natural habitat. By mid-May, mortality in the felt-based system reached approximately 50%, increasing to nearly 90% by June. Consequently, all experimental observations involving the felt-based module were terminated by the end of June due to plant mortality and system failure.

***Comparative Assessment of Abiotic Factors Between Natural and Pot-Based System***

In the experimental phase, the VGS environment was compared with natural ecosystems. Table 3 shows the findings of abiotic variables obtained from monitoring experiments conducted to evaluate the adaptability and potential of S. *resinosum* under cultivation conditions in the pot-based system. Since the same soil was used in the cultivation environment, pH values exhibited similar properties to those of the natural habitat, whereas the other parameters showed distinct differences.

**Table 3:** Observed abiotic variables in the natural habitat and VGS environment of *S. resinosum*.

Months	Natural habitat									VGS Environment								
	Light (lux)	SE	Air_t_ (°C )	SE	Soil_t_ (°C )	SE	Soil_m_ (mm)	SE	Precip. (mm)	Light (lux)	SE	Air_t_ (°C )	SE	Soil_t_ (°C )	SE	Soil_m_ (mm)	SE	Precip. (mm)
March	1250.0	± 9.4	8.2	± 1.1	11.9	± 0.3	50.6	± 3.5	80<	1286.4	± 6.1	10.2	± 1.1	12.6	± 0.5	47.5	± 4.0	80<
April	1469.4	± 14.2	11.4	± 1.9	12.6	± 0.4	45.2	± 1.1	80<	1250.6	± 52.2	13.2	± 1.9	14.7	± 0.8	44.2	± 2.4	80<
May	1053.0	± 17.3	15.4	± 1.1	14.4	± 1.3	39.8	± 0.9	80<	1838.8	± 4.5	17.0	± 1.1	19.7	± 0.4	42.3	± 4.9	15.0
June	1237.6	± 62.4	21.1	± 0.5	18.1	± 0.4	35.4	± 1.2	25.0	1864.8	± 27.5	23.2	± 0.5	23.6	± 1.7	42.7	± 1.2	0.0
July	1395.0	± 30.8	22.2	± 0.7	21.1	± 0.4	32.3	± 1.2	0.0	1907.0	± 29.1	24.4	± 0.7	27.1	± 0.5	44.4	± 1.2	0.0
August	810.0	± 80.9	21.6	± 0.7	27.5	± 0.6	28.5	± 1.8	0.0	1643.2	± 73.5	23.2	± 0.7	27.5	± 0.4	39.7	± 0.9	40.0
September	850.0	± 128.2	18.7	± 1.4	23.5	± 0.9	34.7	± 0.7	0.0	1633.2	± 73.5	20.5	± 1.4	23.3	± 2.1	41.4	± 4.3	0.0
October	202.0	± 12.0	14.3	± 1.9	21.1	± 0.6	43.5	± 0.7	25.0	683.0	± 54.8	16.0	± 1.9	19.1	± 1.6	43.0	± 4.2	80<

*Values are presented as mean ± standard error (SE).* Airt *represents air temperature (°C).* Soilt *represents soil temperature (°C).* Soilm *represents soil moisture (mm). and* Precip. *represents precipitation (mm).*

Light intensity did not differ markedly overall; nevertheless, maximum values were higher in the VGS, reaching up to 1907 lx. Air temperature in the VGS environment was on average approximately 2 °C higher than in the natural habitat during the monitoring period. Differences in soil temperature were more pronounced. In May, soil temperature reached 19.7 ± 0.4 °C in the VGS compared to 14.4 ± 1.3 °C in the natural habitat. This trend continued in June (23.6 ± 1.7 °C vs. 18.1 ± 0.4 °C) and July (27.1 ± 0.5 °C vs. 21.1 ± 0.4 °C). Soil moisture in the natural habitat showed substantial seasonal fluctuation, ranging from 50.6 ± 3.5 mm in March to 28.5 ± 0.9 mm in August, corresponding with precipitation variability. In contrast, soil moisture in the VGS remained relatively stable, ranging between 47.5 ± 4.0 mm and 39.7 ± 1.2 mm, largely independent of external precipitation patterns. Precipitation in the natural habitat was highest in spring (≥ 80 mm), decreasing to moderate levels in June and October (25 mm).

***Comparative Assessment of Biotic Factors Between Natural and Pot-Based System***

The monitoring studies conducted between March and October revealed that the species successfully acclimatized to the VGS during the initial months, with its growth and developmental rates in May closely resembling those observed under natural conditions. Pot-based system observations conducted during July and August revealed that plants in the potted system exhibited approximately 50–60% foliage coverage, and flowering had transitioned into the seed development stage. *S. resinosum* continued to flower throughout July; however, by August, most individuals had completed the flowering phase and initiated seed formation. In the subsequent month of September, plants that had produced seeds began to show visible signs of senescence, including yellowing of the foliage and progressive tissue aging, coinciding with seed maturation. By the end of October, the seeds and flower stalks of the plants had fully desiccated, while the remaining plant tissues within the vertical garden modules exhibited partial drying, indicating the end of the vegetative cycle under ex-situ conditions.

**Fig. 5:**
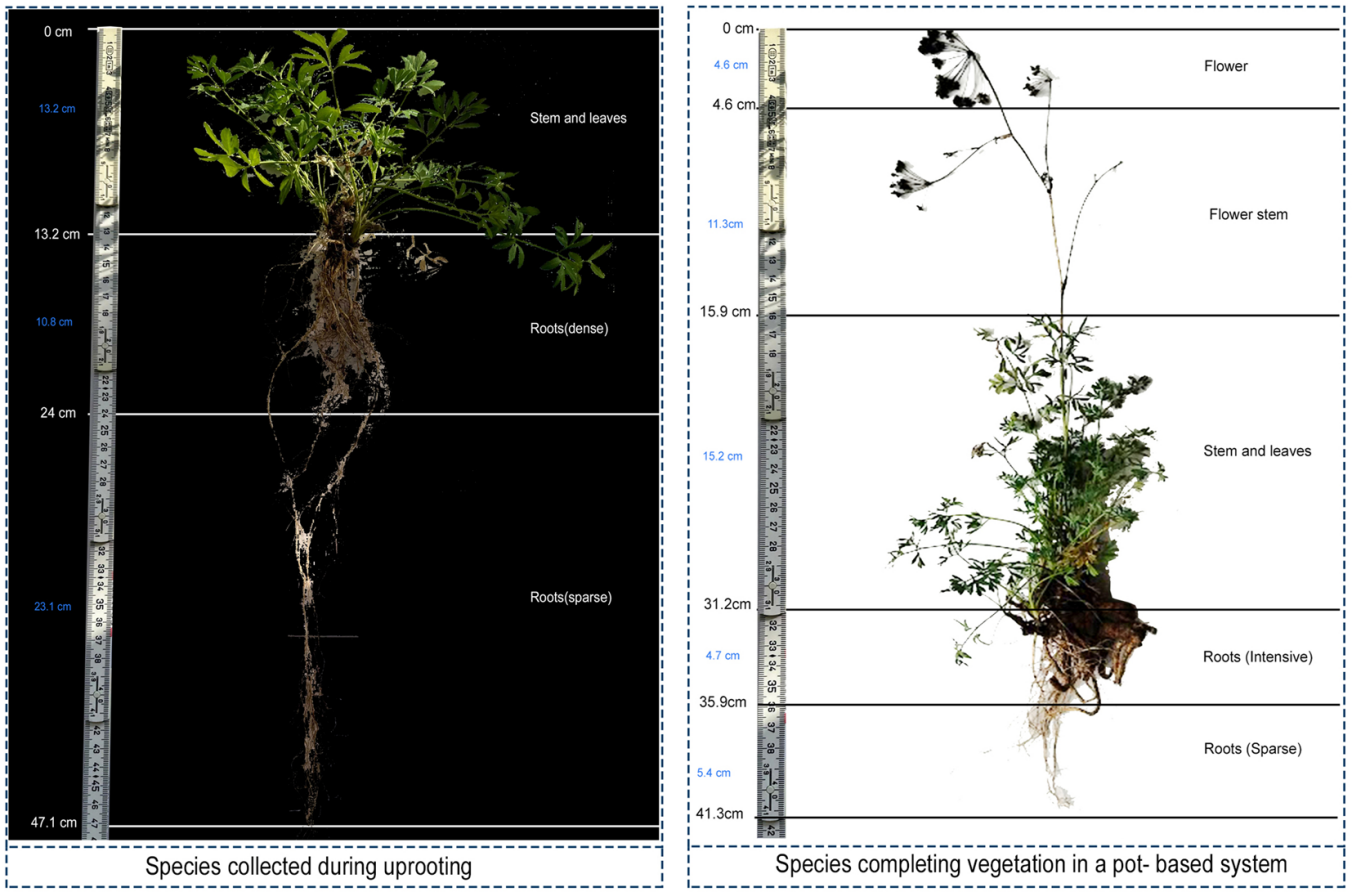
Comparative morphology of *S. resinosum*

According to the morphological measurements conducted to compare the growth performance of *S. resinosum* collected from its natural habitat in March with those cultivated in the potted vertical garden system, the species demonstrated a healthy and sustained vegetative development throughout the growing season (Fig. 5).

*Wild-collected individual (left) exhibited deeper and more differentiated root development (November- measurement)*,* pot-grown individual (right) showed greater aboveground height but reduced root extension (October measurement).*

The overall plant height reached 41.3 cm. When first recorded under natural field conditions, the mean stem height of *S. resinosum* was approximately 13.2 cm. At the end of the vegetative cycle in the pot-based vertical system, mean stem height increased to 15.2 cm, indicating sustained vegetative development under cultivation. Leaf number and overall foliar coverage also increased during this period. The flower zone measured 4.6 cm, resulting in a total reproductive structure height of 15.9 cm. Root architecture differed markedly between environments. The wild-collected specimen exhibited a vertically extensive and structurally differentiated root system reaching 33.9 cm, consisting of a dense root zone (10.8 cm) and a sparse root zone (23.1 cm). In contrast, pot-grown individuals displayed a spatially confined root system, with an intensive root zone of 4.7 cm and a sparse root zone of 5.4 cm, totaling approximately 10.1 cm in root depth. Table 4 compares the vegetative and phenological characteristics of *S. resinosum* under natural habitat conditions and within the VGS throughout the vegetation period.

**Table 4:** Specimen structures in the natural habitat and pot- based system.

Parameter	Natural habitat	VGS (Pot-based system)
Plant height (cm)	39.2 ±5.2	28.4 ±3.84
Pedicel length (cm)	27.3 ± 6.32	20.6 ± 3.17
Number of leaves	21.3 ± 5.39	19.1 ± 6.4
Root length (cm)	34.7 ± 9.3	10,5 ± 5.2
First blooming time	4th week of July	1st week of August
Last blooming time	3st week of September	2rd week of October

Table 4. Specimen structures in the natural habitat and pot-based system.

According to Table 4, plants exhibited relatively similar growth performance in both the natural habitat and the pot-based system. Mean plant height was greater in the natural habitat (39.2 ± 5.2 cm) compared to the pot-based system (28.4 ± 3.84 cm). Similarly, pedicel length was longer under natural conditions (27.3 ± 6.32 cm) than in the VGS environment (20.6 ± 3.17 cm). Leaf number showed moderate variation between environments, with 21.3 ± 5.39 leaves in the natural habitat and 19.1 ± 6.4 leaves in the pot-based system. Phenologically, flowering began earlier and the flowering period ended in the 3rd week of September in the natural habitat. In contrast, in the pot-based system, flowering was delayed, beginning in the last week of July and ending in the second week of October, indicating a shift in reproductive timing under ex situ conditions.

## Discussion

This study represents a novel and integrative approach to biodiversity conservation by combining in situ ecological analysis with ex-situ conservation of *S. resinosum* trials in different VGS. Dahanayake et al.^52^ demonstrated that not every plant species is suitable for all vertical garden systems, as variations in supporting structures and soil depth among VGS designs influence plant adaptability. Most VGS studies^49–52^, focus on ornamental/commercial taxa; our study tests a narrow endemic as ex-situ conservation method.

Ischenko and Shishkunova^62^ stated that the felt-based VGS is the most cost-effective greening solution compared to pot-based systems. Our results revealed that *Seseli resinosum* did not successfully adapt to the felt-based VGS. Similarly, Gür and Kahraman^63^ found that *Lavandula stoechas* L. which is native species in Türkiye exhibited significantly reduced plant diameter when grown in felt-based systems compared to pot based. Similarly, Lavandula species prefer well-drained soils similar to *S. resinosum*^64^. The ecological parallels between these species suggest that felt systems may inherently lack the structural and hydrological conditions necessary to support drought-tolerant endemics, particularly when deployed in outdoor settings without microclimatic buffering. Additionally, Dominici et al.^65^ demonstrated that while felt and pot-based systems are comparable in initial installation costs, the pot system proves significantly more cultivation-friendly in terms of maintenance, irrigation efficiency, and drainage performance. According to Wang et al.^66^ and Sakıcı and Bal^67^, the biophysical properties of the cultivation substrate should closely resemble those of the natural habitat to support optimal growth. Our results parallel those of Dominici et al.^65^, who demonstrated the difficulties of irrigation adjustment in felt-based VGS and by July we lost all the plants due to moisture imbalance. Although both systems were irrigated at equal frequency, the felt substrate exhibited rapid moisture loss, with humidity declining to ~ 40% within three hours after irrigation and drying approximately every two hours during warmer months. In contrast, the pot-based system maintained relatively stable soil moisture levels (42–45%) under the same irrigation regime. Additionally, substrate temperature in the felt system was consistently 2–3 °C higher, likely accelerating evaporation and increasing plant water demand.

However, notable ecological and phenological differences were observed between the ex situ cultivation environment and the natural habitat. Similar results were reported by Yücel and Erken^68^ in their study on the ex situ conservation of the endemic *Campanula grandis* FISCH & C.A. Mey. The study results indicated significant differences in air and soil temperature. The approximately 2 °C difference due to elevation can affect plant growth parameters. Soil temperature differences were particularly pronounced in May, June, and July, when pot-based system showed values ​​4–6 °C higher than natural soils. Such high soil temperatures are known to affect root respiration, enzymatic activity, and water requirements^69,70^.

Owing to the irrigation regime in the pot-based system, soil moisture values remained more stable than those in the natural environment. Nevertheless, such stability may modify the natural growth rhythms of endemic species^71^. Despite the observed abiotic differences, morphological assessments indicated that the plants maintained healthy growth in both environments. The shorter root length observed in the pot-based system may suggest that root development remained incomplete following root pruning at the time of planting. Phenologically, flowering and senescence occurred later in the VGS system compared to the natural habitat. Similarly, Yücel and Erken^68^ reported delayed flowering in the ex situ environment, consistent with the findings of our study. Our results are further supported by Torun and Aydın^56^, who cultivated *S. resinosum* under controlled pot conditions and reported that the vegetation period of the control group progressed normally. In a study conducted by Aydın et al.^72^, *S. resinosum* was successfully germinated in a controlled environment containing 50% cocopeat (coconut fiber) and 50% perlite for one month under day–night cycles at 60–70% relative humidity. Following germination, seedlings were cultivated in a substrate composed of peat, perlite, and river sand in a 1:1:1 ratio. Despite these standardized horticultural conditions, the plant’s growth and development were reported to be inferior to those observed in its natural habitat. The morphological development observed in our study also aligns closely with natural benchmarks reported in the literature. Eroğlu et al. (2019) documented that *S. resinosum* typically reaches heights of 30–50 cm in its native habitat. In our modular potted vertical garden system, the species exhibited growth ranging from 19.1 ± 6.4 cm. Eroğlu and Başaran (2017), Sakıcı and Bal^68^, and Meral et al.^48^, all highlight the ecological advantages of using native flora in VGS implementations. Ottelé et al.^53^ emphasized that the coexistence of species exhibiting similar ecological traits enhances their adaptation success in vertical garden systems. *S. resinosum* exhibits similar ecological traits to *H. perforatum*, *O.vulgare*, *S. italica*, and *S. columbaria*.

### Limitations and Future Research

Despite the successful adaptation observed in the pot-based VGS, the study was subject to several limitations. First, the study evaluated a single vegetation cycle; long-term survival, reproductive success, and seed viability under VGS conditions remain unknown. Second, due to the limited number of collected individuals, biotic measurements could not be conducted on a monthly basis. Third, scaling up the system for conservation programs would require assessment of genetic diversity maintenance, population stability, and species composition in association with co-occurring native taxa.

Future research should focus on multi-year monitoring, reproductive output quantification, and testing additional endemic or native taxa with varying ecological strategies. Integrating physiological measurements such as water potential, stomatal conductance would further clarify mechanistic drivers of adaptation.

## Conclusion

This study fills a significant research gap by integrating ecological field assessment with experimental cultivation to evaluate the survival and morphological performance of *S. resinosum* an endemic species from the Düzce Province of Türkiye. The species’ natural habitat, defined by red-brown forest soils with shallow depth, low organic matter, and xeric moisture levels, points to a high degree of habitat specialization. Field observations confirmed its preference for rocky, steep-slope environments within landslide zones and its tendency to grow in dense colonies.

In the felt-based system, substrate moisture declined to ~ 40% within 3 h after irrigation and dried approximately every 2 h during warm periods. Substrate temperature remained 2–3 °C higher than in the pot-based system. Mortality reached ~ 50% by mid-May and ~ 90% by June, with complete plant loss occurring within 3.5 months. In contrast, the pot-based system (8 cm substrate depth) maintained stable soil moisture (42–45%) under once-daily irrigation and supported a complete vegetative and reproductive cycle. Plants reached a mean height of 28.4 ± 3.84 cm and successfully completed flowering and seed development. These results demonstrate that substrate volume, hydrological buffering capacity, and thermal regulation are critical determinants of ex situ survival for endemic species.

In addition, *S. resinosum* shares ecological traits with other taxa such as *H. perforatum*, *O. vulgare*, *S. italica*, and *S. columbaria*, suggesting that future vertical garden designs incorporating complementary species compositions may further enhance habitat simulation and adaptation success. In future research, the preparation of vertical garden compositions with species sharing similar traits to *S. resinosum* could play a key role in replicating analogous habitat conditions, thereby enhancing the effectiveness of ex situ conservation studies.

In conclusion, this research provides empirical evidence that modular pot-based VGS can be integrated into urban green infrastructure as a novel ex situ conservation strategy for endemic flora.

## Data Availability

The datasets generated and analyzed during the current study are available from the corresponding author upon reasonable request.

## References

[1] IUCN & Background & history. (2024). https://www.iucnredlist.org/about/background-history

[2] Lacher, T. E. Jr et al. The status, threats and conservation of critically endangered species. *Nat. Rev. Biodivers.* **1**, 1–18. 10.1038/s44358-025-00059-4 (2025).

[3] Kraus, D. et al. Prioritizing nationally endemic species for conservation. *Conserv. Sci. Pract.* **5**, e12845. 10.1111/csp2.12845 (2023).

[4] Wagensommer, R. P., Medagli, P., Turco, A. & Perrino, E. V. IUCN Red List evaluation of the Orchidaceae endemic to Apulia region (Italy) and considerations on the application of the IUCN protocol to rare species. *Nat. Conserv. Res.* **5** (Suppl. 1), 1–5. 10.24189/ncr.2020.033 (2020).

[5] Ben Mahmoud, K., Mezzapesa, G. N., Abdelkefi, F. & Perrino, E. V. Nutritional value and functional properties of an underexploited Tunisian wild beet (Beta macrocarpa Guss.) in relation to soil characteristics. *Euro-Mediterr J. Environ. Integr.* **9**10.1007/s41207-024-00468-5 (2024).

[6] Pritchard, D. J., Fa, J. E., Oldfield, S. & Harrop, S. R. Bring the captive closer to the wild: redefining the role of ex situ conservation. *Oryx* **46**, 18–23 (2012).

[7] Awazi, N. P., Ambebe, T. F. & Njamnjubo, N. A. Ex-situ conservation of threatened flora and fauna species in Cameroon through botanic and zoological gardens: governance and policy paradigms. *Discover Conserv.* **2**, 26. 10.1007/s44353-025-00049-9 (2025).

[8] Engelmann, F. Use of biotechnologies for the conservation of plant biodiversity. *Vitro Cell. Dev. Biol. –Plant*. **47**, 5–16. 10.1007/s11627-010-9327-2 (2011).

[9] Perrino, E. V., Wagensommer, R. P., Mezzapesa, G. N. & Trani, A. Stachys italica Mill.: synecology, functional compounds and potential use of an Italian endemic taxon. *Planta* **260**, 138. 10.1007/s00425-024-04571-3 (2024). 39545970 10.1007/s00425-024-04571-3

[10] Reed, B. M., Sarasan, V., Kane, M., Bunn, E. & Pence, V. C. Biodiversity conservation and conservation biotechnology tools. *Vitro Cell. Dev. Biol. –Plant*. **47**, 1–4. 10.1007/s11627-010-9337-0 (2011).

[11] Werden, L. K. et al. Ex situ conservation of threatened plant species in island biodiversity hotspots: a case study from Hawai‘i. *Biol. Conserv.* **243**, 108435. 10.1016/j.biocon.2020.108435 (2020).

[12] Burlakova, L. E. et al. Endemic species: contribution to community uniqueness, effect of habitat alteration, and conservation priorities. *Biol. Conserv.* **144**, 155–165. 10.1016/j.biocon.2010.08.010 (2011).

[13] Salariato, D. L., Zanotti, C. & Zuloaga, F. O. Threat patterns for endemic plants of Argentina reveal disparity of vulnerability and protection among spatially associated species groups. *J. Nat. Conserv.* **74**, 126422. 10.1016/j.jnc.2023.126422 (2023).

[14] Aksoy, N., Özkan, N. G., Aslan, S. & Koçer, N. Plant biodiversity, endemic and rare plant taxa and their conservation status in Düzce province [in Turkish]. In Düzce’de Tarih ve Kültür (ed Ertuğrul, A.) 361–375 (Gaye, (2014).

[15] Li, L., Huang, X., Wu, D. & Yang, H. Construction of ecological security pattern adapting to future land use change in Pearl River Delta. *China Appl. Geogr.* **154**, 102946. 10.1016/j.apgeog.2023.10294 (2023).

[16] Perrino, E. V., Zdruli, P., Piscitelli, L. & D’Agostino, D. Restoration, indicators, and participatory solutions: addressing water scarcity in Mediterranean agriculture. *Agronomy* **15**, 1517–1533. 10.1007/s00425-024-04571-3 (2025).

[17] Coelho, N., Gonçalves, S. & Romano, A. Endemic plant species conservation: biotechnological approaches. *Plants* **9**, 345. 10.3390/plants9030345 (2020). 32182892 10.3390/plants9030345 PMC7154900

[18] Perrino, E. V. & Wagensommer, R. P. Crop wild relatives (CWR) priority in Italy: distribution, ecology, in situ and ex situ conservation and expected actions. *Sustainability* **13**, 168. 10.3390/su13041682 (2021).

[19] Zegeye, H. In situ and ex situ conservation: complementary approaches for maintaining biodiversity. *Int. J. Res. Environ. Stud.* **4**, 1–12 (2017).

[20] Tas, N. et al. Conservation gap analysis of crop wild relatives in Turkey. *Plant. Genet. Resour.* **17**, 164–173. 10.1017/S1479262118000564 (2019).

[21] Duman, H., Doğan, M., Atlı, Ö. & Celep, F. Ex situ and in situ conservation approaches in species-rich Anatolian steppe ecosystem: a case study from Ankara. *Türkiye Ecologies*. **5**, 664–678. 10.3390/ecologies5040039 (2024).

[22] Noroozi, J. et al. Patterns of endemism in Turkey, the meeting point of three global biodiversity hotspots, based on three diverse families of vascular plants. *Front. Ecol. Evol.* **7**, 159. 10.3389/fevo.2019.00159 (2019).

[23] Uludağ, A. et al. Alien flora of Turkey: checklist, taxonomic composition and ecological attributes. *NeoBiota* **35**, 61–85. 10.3897/neobiota.35.12460 (2017).

[24] Aksoy, N., Özkan, N. G., Koçer, N., Müderrisoğlu, H. & Eroğlu, E. Risk management plan for natural and exotic aquatic plant species in Bolu Gölcük [in Turkish]. *Anatol. J. Res.* **9**, 171–182. 10.53516/ajfr.1266251 (2023).

[25] Kaya, S., Eroğlu, E., Başaran, N., Ayteğin, A. & Dönmez, A. H. Determination of the natural plant compositions and species distribution model in different habitat types of Düzce (Türkiye). *Cerne* **31**, e–103449. 10.1590/01047760202531013449 (2025).

[26] Eroğlu, E. et al. Determination of the visual preferences of different habitat types. *Fresenius Environ. Bull.* **27**, 4889–4899 (2018).

[27] Aydın, H., Torun, H. & Eroğlu, E. Morphological and physiological characteristics and landscape use potential of the endemic taxa Cephalaria duzceënsis N. Aksoy & R. S. Göktürk and Seseli resinosum Freyn & Sint. *Düzce Univ. J. For.* **16**, 89–104 (2020).

[28] Aksoy, N. & Uzun, O. Distribution and conservation significance of endemic plants in the Düzce province. *Int. J. Phys. Sci.* **6**, 2143–2151. 10.5897/IJPS11.194 (2011).

[29] Heywood, V. H. An overview of in situ conservation of plant species in the Mediterranean. *Flora Mediterr.* **24**, 5–24. 10.7320/FlMedit24.005 (2014).

[30] Radomir, A. M. et al. Overview of the success of in vitro culture for ex situ conservation and sustainable utilization of endemic and subendemic native plants of Romania. *Sustainability* **15**, 2581. 10.3390/su15032581 (2023).

[31] Hawkes, J. G., Maxted, N. & Ford-Lloyd *B. V. The ex situ conservation of plant genetic resources* (Springer Science & Business Media, 2012).

[32] Rajpurohit, D. & Jhang, T. In situ and ex situ conservation of plant genetic resources and traditional knowledge. In Plant Genetic Resources and Traditional Knowledge for Food Security (eds (eds Salgotra, R. & Gupta, B.) 137–162 (Springer, Singapore, ; 10.1007/978-981-10-0060-7_8. (2015).

[33] Pence, V. C. et al. Defining exceptional species—A conceptual framework to expand and advance ex situ conservation of plant diversity beyond conventional seed banking. *Biol. Conserv.* **266**, 109440. 10.1016/j.biocon.2021.109440 (2022).

[34] Pence, V. C. In vitro methods and the challenge of exceptional species for target 8 of the global strategy for plant conservation. *Ann. Mo Bot. Gard*. **99**, 214–220. 10.3417/2011112 (2013).

[35] Liu, U., Breman, E., Cossu, T. A. & Kenney, S. The conservation value of germplasm stored at the millennium seed bank, Royal Botanic Gardens, Kew, UK. *Biodivers. Conserv.* **27**, 1347–1386. 10.1007/s10531-018-1497-y (2018).

[36] Van Kleunen, M. et al. The changing role of ornamental horticulture in alien plant invasions. *Biol. Rev.* **93**, 1421–1437. 10.1111/brv.12402 (2018). 29504240 10.1111/brv.12402

[37] Manickathan, L. et al. Integrated vegetation model for studying the cooling potential of trees in urban street canyons. *Bound. -Layer Meteorol.* **166**, 301–328. 10.1007/s10546-017-0305-3 (2018).

[38] Rouichi, S., Ghanem, M. E. & Amri, M. In situ and ex situ conservation priorities and distribution of lentil wild relatives under climate change: a modelling approach. *J. Appl. Ecol.* **62**, 414–428. 10.1111/1365-2664.14842 (2025).

[39] Mounce, R., Smith, P. & Brockington, S. Ex situ conservation of plant diversity in the world’s botanic gardens. *Nat. Plants*. **3**, 795–802. 10.1038/s41477-017-0019-3 (2017). 28947807 10.1038/s41477-017-0019-3

[40] Zhao, X. et al. Ex situ conservation of threatened higher plants in Chinese botanical gardens. *Glob Ecol. Conserv.* **38**10.1016/j.gecco.2022.e02206 (2022). e02206.

[41] Ucella-Filho, J. G. M. et al. B. D. Biodiverse neighborhoods: an ex situ conservation tool. *Ornam. Hortic.* **28**, 8–18 (2022).

[42] Davis, M., González-Laprea, J., González, L. J. B. & Ramírez, R. F. Beyond green facades: taking a closer look at the role of endemic plants in vertical gardens. In International Conference on Urban Planning and Architectural Design for Sustainable Development 135–142Springer Nature Switzerland, Cham, (2023).

[43] Katoch, K., Dubey, R. K. & Choudhary, A. Evaluation of different combinations of potting media and ornamental plants on growth, biochemical, and nutrient content in outdoor vertical gardening. *J. Plant. Nutr.* **47**, 3455–3468. 10.1080/01904167.2024.2380480 (2024).

[44] Hindle, R. L. A vertical garden: origins of the vegetation-bearing architectonic structure and system Stud. Hist. Gard. Des. Landsc. 32, 99–110 (2012). (1938). 10.1080/14601176.2011.653535

[45] Sunil, B., Prasanth, P., Salma, Z., Jyothi, G. & Kumar, P. Influence of light intensity on morphological and growth responses of ornamental foliage plants in vertical garden systems. *J. Adv. Biol. Biotechnol.* **28**, 1260–1276. 10.9734/jabb/2025/v28i113318 (2025).

[46] Campiotti, C. A. et al. Vertical greenery as natural tool for improving energy efficiency of buildings. *Horticulturae* **8**, 526. 10.3390/horticulturae8060526 (2022).

[47] Trkulja, T., Radujković, M. & Nikolić-Topalović, M. Vertical greenery system: a model for improving energy efficiency of buildings. *Građevinar* **74**, 561–571. 10.14256/JCE.3370.2021 (2022).

[48] Meral, A. et al. A comparative approach to artificial and natural green walls according to ecological sustainability. *Sustainability* **10**, 1995. 10.3390/su10061995 (2018).

[49] Eroğlu, E. & Başaran, N. Evaluation of visual landscape quality of indoor vertical garden plant compositions. *Düzce Univ. J. For.* **13**, 32–49 (2017).

[50] Kaltsidi, M. P., Bayer, I., Mitsi, C. & Aros, D. Potential use of Chilean native species in vertical greening systems. *Sustainability* **15**, 4944. 10.3390/su15064944 (2023).

[51] Sun, Q. et al. Differences in ecological traits between plants grown in situ and ex situ and implications for conservation. *Sustainability* **14**, 5199. 10.3390/su14095199 (2022).

[52] Dahanayake, K. C., Chow, C. L. & Hou, G. L. Selection of suitable plant species for energy efficient vertical greenery systems (VGS). *Energy Procedia*. **142**, 2473–2478. 10.1016/j.egypro.2017.12.185 (2017).

[53] Ottelé, M., Perini, K., Fraaij, A. L. A., Haas, E. M. & Raiteri, R. Comparative life cycle analysis for green façades and living wall systems. *Energy Build.* **43**, 3419–3429 10.1016/j.enbuild.2011.09.010 (2011).

[54] Manso, M., Castro-Gomes, J., Paulo, B., Bentes, I. & Teixeira, C. A. Life cycle analysis of a new modular greening system. *Sci. Total Environ.* **627**, 1146–1153. 10.1016/j.scitotenv.2018.01.198 (2018). 29426132 10.1016/j.scitotenv.2018.01.198

[55] Reyhani, M., Santolini, E., Torreggiani, D. & Tassinari, P. Assessing the environmental performance of plastic-based and felt-based green wall systems in a life-cycle perspective. *Sci. Total Environ.* **822**, 153648. 10.1016/j.scitotenv.2022.153648 (2022). 35124065 10.1016/j.scitotenv.2022.153648

[56] Torun, H. & Aydın, H. Physiological and antioxidative responses of the endemic plant Seseli resinosum Freyn & Sint. to drought stress. *Düzce Univ. J. Forestry*. **17** (1), 199–213 (2021).

[57] Eroğlu, E. et al. Determination of seasonal change potential of some endemic plant species in Düzce and its surrounding area. *Düzce Univ. J. Sci. Technol.* **7**, 1686–1697. 10.29130/dubited.551678 (2019).

[58] Turkish State Meteorological Service (TSMS). Forecast cities: Düzce. (2025). http://www.mgm.gov.tr/eng/forecast-cities.aspx?m=DUZCE

[59] Braun-Blanquet, J. *Plant sociology: The study of plant communities* (McGraw-Hill, 1932).

[60] Ministry of Agriculture and Forestry. Official Gazette (2024). https://www.resmigazete.gov.tr/eskiler/2024/09/20240917-2.htm

[61] Real, R. & Vargas, J. M. The probabilistic basis of Jaccard’s index of similarity. *Syst. Biol.* **45**, 380–385 (1996). https://www.jstor.org/stable/2413572

[62] Ischenko, A. & Shishkunova, D. Application of vertical gardening technology in high-rise construction. *E3S Web Conf.* **258** (09035). 10.1051/e3sconf/202125809035 (2021).

[63] Gür, N. & Kahraman, Ö. Usability of Lavandula stoechas in some vertical garden systems. *Int. J. Landsc. Archit. Res.* **5**, 1–10 (2021).

[64] Başaran, N. Bringing lavender to economy in rural development and rural tourism scope. *Res. J. Agric. Sci.* **10**, 47–49 (2017).

[65] Dominici, L., Comino, E., Torpy, F. & Irga, P. Vertical greening systems: a critical comparison of do-it-yourself designs. *Plants* **11**, 3230. 10.3390/plants11233230 (2022). 36501270 10.3390/plants11233230 PMC9739368

[66] Wang, C., Wood, L. C. & Teo, L. T. Tropical vertical greenery systems: irrigation systems, biophysical characteristics, and influential criteria. *J. Green. Build.* **11**, 57–90 (2016).

[67] Sakıcı, Ç. & Bal, A. N. Determination of use of some natural plant species in vertical garden systems in İzmir region. *Düzce Univ. J. For.* **18**, 226–246 (2022).

[68] Yücel, G. & Erken, K. Optimal germination methods, ornamental plant features, and ex situ conservation of endemic Campanula grandis Fisch. *C Mey J. Environ. Eng. Landsc. Manag*. **31**, 132–141. 10.3846/jeelm.2023.19018 (2023).

[69] Rahman, M. T., Zhu, Q. H., Zhang, Z. B., Zhou, H. & Peng, X. The roles of organic amendments and microbial community in the improvement of soil structure of a Vertisol. *Appl. Soil. Ecol.* **111**, 84–93. 10.1016/j.apsoil.2016.11.018 (2017).

[70] Bogati, K. & Walczak, M. The impact of drought stress on soil microbial community, enzyme activities and plants. *Agronomy* **12**, 189. 10.3390/agronomy12010189 (2022).

[71] Rauschkolb, R., Szczeparska, L., Kehl, A., Bossdorf, O. & Scheepens, J. F. Plant populations of three threatened species experience rapid evolution under ex situ cultivation. *Biodivers. Conserv.* **28**, 3951–3969. 10.1007/s10531-019-01859-9 (2019).

[72] Aydın, H., Torun, H., Eroğlu, E. & Aksoy Morphological and physiological characteristics and landscape use potential of the endemic taxa Cephalaria duzceënsis N. & R. S. Göktürk and Seseli resinosum Freyn & Sint. Düzce Univ. J. For. 16, 89–104 (2020).

